# Thermally robust perpendicular Co/Pd-based synthetic antiferromagnetic coupling enabled by a W capping or buffer layer

**DOI:** 10.1038/srep21324

**Published:** 2016-02-18

**Authors:** Ja-Bin Lee, Gwang-Guk An, Seung-Mo Yang, Hae-Soo Park, Woo-Seong Chung, Jin-Pyo Hong

**Affiliations:** 1 Novel Functional Materials and Device Lab., Research Institute of Convergence of Basic Science, Department of Physics, Hanyang University, Seoul, 133-791, South Korea; 2 Nano Quantum Electronics Lab., Department of Electronics and Computer Engineering, Hanyang University, Seoul, 133-791, South Korea

## Abstract

Perpendicularly magnetized tunnel junctions (p-MTJs) that contain synthetic antiferromagnetic (SAF) frames show promise as reliable building blocks to meet the demands of perpendicular magnetic anisotropy (PMA)-based spintronic devices. In particular, Co/Pd multilayer-based SAFs have been widely employed due to their outstanding PMA features. However, the widespread utilization of Co/Pd multilayer SAFs coupled with an adjacent CoFeB reference layer (RL) is still a challenge due to the structural discontinuity or intermixing that occurs during high temperature annealing. Thus, we address the thermally robust characteristics of Co/Pd multilayer SAFs by controlling a W layer as a potential buffer or capping layer. The W-capped Co/Pd multilayer SAF, which acts as a pinning layer, exhibited a wide-range plateau with sharp spin-flip and near-zero remanence at the zero field. Structural analysis of the W-capped multilayer SAF exhibited single-crystal-like *c*-axis oriented crystalline features after annealing at 400 °C, thereby demonstrating the applicability of these frames. In addition, when the W layer serving as a buffer layer in the Co/Pd multilayer SAF was coupled with a conventional CoFeB RL, higher annealing stability up to 425 °C and prominent antiferromagnetic coupling behavior were obtained.

Spin-transfer-torque magnetic random access memories (STT-MRAMs) have garnered considerable interest as one of the most attractive candidates to meet the demands of non-volatile memory markets due to their low power consumption, practically unlimited endurance, and sub-20-nm downsize scalability^1^,^2^,^3^. Therefore, to facilitate the development of STT-MRAMs, perpendicularly magnetized magnetic tunnel junctions (*p*-MTJs) have mainly been employed as promising building blocks to ensure outstanding performance. Suitable *p*-MTJ devices consist of diverse multi-layers, including a magnetically soft storage layer, which is separated from a magnetically hard reference layer (RL) by an ultrathin MgO tunnel barrier. In basic *p*-MTJ operation, the magnetization direction of the storage layer should switch to a data storage state of ‘0’ or ‘1’, while the stationary magnetization direction of the RL must be maintained. Therefore, the switching field of the RL must be as high as possible to reduce the chance of causing a switching disturbance. However, the continued miniaturization of *p*-MTJs inherently increases the magnetic stray field arising from the RL^4^,^5^, leading to the presence of an offset field in storage layer switching, which causes a shift from the zero field in the magnetoresistance curve. Therefore, the ability to manipulate the stray field is a key step towards extending their use.

Much effort has been devoted towards the use of a synthetic antiferromagnetic (SAF) frame as a pinning layer, based on interlayer exchange coupling (IEC)^6^,^7^,^8^. SAFs are composed of two ferromagnetic layers with perpendicular magnetic anisotropy (PMA) through a thin spacer layer (e.g., Ru, Rh, or Ir)^9^,^10^,^11^. It is widely believed that the IEC energy density (-*J*_*iec*_) can be modulated by adjusting the spacer layer thickness or the type of insertion layer at the interface of the spacer layer^6^,^11^,^12^. Among various SAF coupling frames that have been considered recently, Co-based multilayers have emerged as a promising alternative for use due to their simple tunability and strong PMA characteristics. Alternating two layers with different thicknesses and selecting the proper repeat number can enable the use of Co-based multilayers in SAF frames. However, the widespread use of Co-based multilayer SAFs, coupled with an adjacent conventional CoFeB RL, remains as a challenge because of the presence of structure discontinuity or intermixing, which occurs during high temperature annealing. Thus, a recent approach included the incorporation of a proper capping layer as a potential solution to prevent unintended diffusion upon annealing. In addition, since the capping layer used for the SAF frame inherently serves as a buffer layer (BL) for the adjacent CoFeB layer^13^,^14^, the choice of a capping or buffer layer must include a proper boron affinity, crystal structure continuity, and/or annealing stability to prevent possible diffusion towards the adjacent CoFeB layer or Co-based multilayers^15^.

Since the discovery of interface PMA in the Ta/CoFeB/MgO structure^16^, numerous reports have been published on the successful development of SAF/Ta/CoFeB RL frames containing a Ta layer^14^,^17^,^18^. However, employing a Ta layer as a generic buffer or capping layer has the disadvantage of rapid PMA degradation, which occurs during annealing at temperatures greater than 300 °C^19^,^20^,^21^. Thus, obtaining an alternative layer that ensures thermally-robust PMA characteristics in Co-based multilayer SAF frames would facilitate the development of fully stacked *p*-MTJs because the back-end-of-line progress requires a process temperature of 350 °C or higher^22^.

This letter addresses the influence of using a W layer as a capping and buffer layer (BL) on the Co/Pd multilayer SAF and CoFeB RL, respectively. Magnetic responses at different W thicknesses and annealing conditions were systematically tested. Experimental findings verified the achievement of distinct antiferromagnetic coupling (AFC) features with a sharp spin-flip in the Co/Pd multilayer SAF frames up to 425 °C, thereby validating their ability to be used for real device applications. Structural analysis of the W-capped multilayer SAF showed single-crystal-like *c*-axis oriented crystalline features after annealing at 400 °C. In addition, the introduction of a suitable W layer as a BL significantly enhances the PMA characteristics and AFC features of CoFeB RL frames, helping them to meet the demands of enhanced output performance.

## Results

### Ru spacer thickness optimization

**Figure 1:**
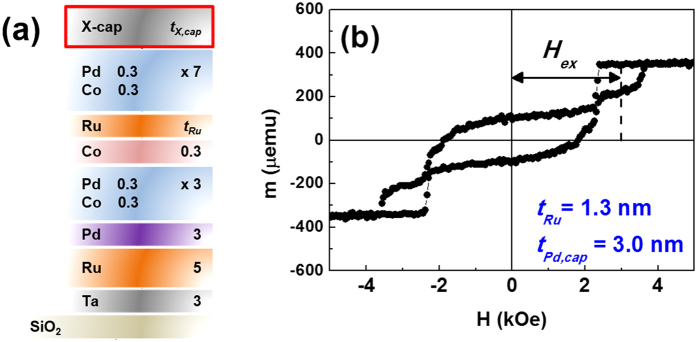
(**a**) Schematic illustration of the X-capped SAF structures. (**b**) Representative M-H hysteresis loop of as-deposited Pd-capped SAF with Ru spacer thickness of *t*_*Ru*_ = 1.3 nm. Exchange coupling field (*H*_*ex*_) is defined as minor loop shift.

The SAF frames were prepared as follows: subs./Ta (3)/Ru (5)/Pd (3)/[Co (0.3)/Pd (0.3)]_3_/Co (0.3)/Ru (*t*_*Ru*_)/[Co (0.3)/Pd (0.3)]_7_/X-capping (*t*_*X,cap*_) (hereafter referred to as X-capped SAF). The numbers in the parentheses refer to the nominal thicknesses in nanometers and the subscripts for the Co/Pd multilayers refer to the stacking repetition numbers. Figure 1(a) shows the representative schematic for the X-capped SAF structure. It is widely believed that the performance of the SAF depends on the perpendicular exchange coupling field (*H*_*ex*_), which is defined as the field shift of the minor loop. Thus, the magnetic responses of the SAF were first recorded as a function of the Ru spacer thickness (varying in the range of 0.3 ≤ *t*_*Ru*_ ≤ 2.0 nm) with a fixed Pd capping layer (thickness of *t*_*Pd,cap*_ = 3 nm). Figure 1(b) displays the representative magnetic hysteresis (M-H) loop for the Pd-capped SAF with Ru spacer thickness of *t*_*Ru*_ = 1.3 nm, in which an exchange coupling field *H*_*ex*_ is denoted as a minor loop shift. More detailed individual M-H loop, *H*_*ex*_, and calculated -*J*_*iec*_ values are given in Figure S1 of the Supporting Information. In addition, a Pd layer was chosen as a capping layer to determine the optimum Ru thickness in the SAF; our previous work on Pd-capped Co/Pd multilayers verified high annealing stability at above 400 °C^23^. The Pd-capped SAFs with *t*_*Ru*_ = 1.3 nm exhibited net magnetization reversal with maximum *H*_*ex*_ and -*J*_*iec*_ values of 2.97 kOe and 0.66 erg/cm^2^, respectively. The -*J*_*iec*_ was determined by using the equation of -*J*_*iec*_ = *H*_*ex*_*M*_*S*_*t*_*FM*_, where *M*_*S*_ and *t*_*FM*_ refer to the saturation magnetization and total thickness of the Co/Pd multilayers, respectively. Previous work by Parkin *et al.* indicated that the SAF with a Ru spacer provided an oscillatory decay motion with the highest -*J*_*iec*_ at the first peak of *t*_*Ru*_ (around 0.4 nm)^11^. However, several other studies also showed moderate AFC behavior at the second peak of oscillation^24^,^25^,^26^. Similarly, the Pd-capped SAF demonstrated the highest -*J*_*iec*_ at the second peak of nominal *t*_*Ru*_ (at 1.3 nm). This is likely attributed to the rough or unclear interface of the ultrathin Ru spacer, resulting in inadequate AFC behavior. However, the relatively thick *t*_*Ru*_ in our work may lead to a distinct and strong AFC, which may be induced by the formation of a relatively uniform and clear Ru interface. Therefore, a 1.3-nm-thick Ru spacer layer was experimentally selected for the following measurements.

### Capping layer effect

**Figure 2:**
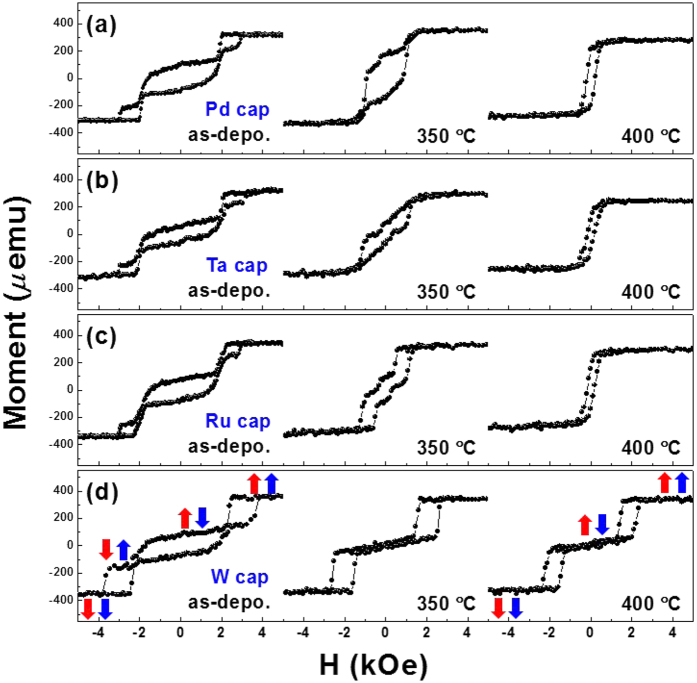
Typical M-H hysteresis loops of SAFs with (**a**) Pd, (**b**) Ta, (**c**) Ru, and (**d**) W capping layers annealed at high temperatures of 350 °C and 400 °C for 1 h under 3 Tesla. Red and blue arrows in the figures represent the magnetization direction of the upper (repetition number = 7) and lower (repetition number = 3) portions of the Co/Pd multilayers, respectively.

Figure 2 presents the representative M-H loops of Pd-, Ta-, Ru-, and W-capped SAFs annealed at 350 and 400 °C, where various metal layers were chosen as capping layers for comparison. The W layer was also included in this measurement because the W layer exhibited outstanding PMA features in the W/CoFeB/MgO frame containing a W seed layer^27^. As shown in this figure, all samples in the as-deposited state displayed similar M-H shapes, implying net magnetization reversal without breaking the AFC. Previous works have demonstrated that both the observed M-H loop shape of AFC and its switching nature rely on the ratio between -*J*_*iec*_ and the areal effective PMA energy density (*K*_*eff*_ · *t*_*FM*_)^28^,^29^. If -*J*_*iec*_ is less than *K*_*eff*_ · *t*_*FM*_ (-*J*_*iec*_ < *K*_*eff*_ · *t*_*FM*_), a wide range plateau appears over the whole AFC region. Meanwhile, when the IEC energy is comparable to or exceeds the PMA energy (-*J*_*iec*_ ≥ *K*_*eff*_ · *t*_*FM*_), the aforementioned plateau becomes narrower and results in another simultaneous spin-flip of two ferromagnetic layers with head-to-head and tail-to-tail magnetic configurations. Further detailed descriptions for various spin-flip events are presented in Figure S2 of the Supporting Information, along with typical schematics. In our case, for example, the maximum -*J*_*iec*_ of as-deposited Pd-capped SAF was about 0.66 erg/cm^2^ for *t*_*Ru*_ = 1.3 nm, which exceeds a *K*_*eff*_ · *t*_*FM*_ value of Co/Pd multilayers with a repetition number of 10 (0.63 erg/cm^2^). The *K*_*eff*_ value was determined from the enclosed area between the in-plane and out-of-plane M-H curves. Thus, the trend of our as-deposited SAFs agreed with the results observed for the well-known -*J*_*iec*_ ≥ *K*_*eff*_ · *t*_*FM*_ case. Further detailed *H*_*ex*_, *K*_*eff*_ · *t*_*FM*_ and -*J*_*iec*_ information for Fig. 2 are given in the Table 1. However, annealing allows for the development of different behaviors among various capping layers. For example, it can be seen that the IEC strength of Pd-, Ta-, and Ru-capped SAFs was weakened at 350 °C, resulting in a canted shape with a quite small *H*_*ex*_. Annealing at 400 °C caused the transition from AFC to FC with a slight decrease in the *M*_*S*_. Alternatively, the W-capped SAF exhibited enhanced annealing stability up to 400 °C, as shown in Fig. 2(d). In addition, a wide range AFC plateau with sharp spin-flip appeared and the net magnetization reversal disappeared. This trend is predominantly related to the enhanced PMA features of Co/Pd multilayers created by the crystal structure ordering during annealing^30^. Furthermore, the presence of near-zero remanence at the zero field is likely linked with the formation of additional Co/Pd multilayers upon annealing; this will be discussed later in structural analysis. To further verify the impact of W capping layer, other capping layers (Ru/Ta, Ru/W, and W/Ru/W) were also employed (see Figure S3 of the Supporting Information). The other capping layers were tested in an attempt to confirm whether thermally stable IEC feature of W-capped SAF arose from only W interface or not. In addition, it is widely believed that a Ru material has its superior *c*-axis orientation. Thus, if thermally stable IEC feature completely is governed by only W interface in a W/Ru/W capped frame without being affected by the Ru, a W/Ru/W layer may become one of promising options for use as a buffer layer between SAF structure and CoFeB/MgO junction. The Ru/Ta- and Ru/W-capped SAFs displayed quite similar behaviors observed from the Ru-capped SAF frame. However, the W/Ru/W-capped SAF still maintained typical AFC after annealing at 400 °C, along with the appearance of a canted shape in the AFC region. The Ru diffusion process upon annealing seems contribute to the observed canted M-H shape by affecting the PMA properties of Co/Pd multilayers. Therefore, a single W capping layer seems to serve to provide the optimized SAF frame in our work. Further detailed information for *H*_*ex*_, *K*_*eff*_ · *t*_*FM*_ and -*J*_*iec*_ values observed from Figure S3 is presented in the Table S1.

**Figure 3:**
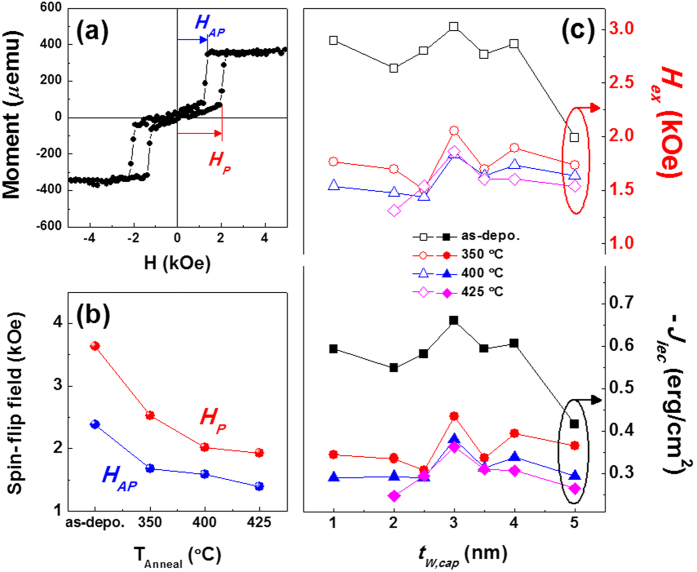
(**a**) Typical M-H loop of W-capped SAF annealed at 425 °C. The spin-flip fields from the parallel to antiparallel configurations and from the antiparallel to parallel configurations between the upper (repetition number = 7) and lower (repetition number = 3) portions of Co/Pd multilayers are defined as *H*_*AP*_ and *H*_*P*_, respectively. (**b**) Plotted *H*_*AP*_ and *H*_*P*_ as a function of the annealing temperature T_Anneal_. (**c**) Plots of the *H*_*ex*_ (top) and -*J*_*iec*_ (bottom) values of W-capped SAFs as a function of the W capping layer thickness.

To further exploit the above observations regarding the effect of the W capping layer, the effect of the W capping layer thickness on the magnetic properties was recorded. Figure 3(a) shows a representative M-H loop of the 3.0-nm-thick W-capped SAF annealed at 425 °C. The wide range AFC plateau with sharp spin-flip was kept, thereby ensuring thermally-stable IEC features. In this figure, spin-flip fields from the parallel (antiparallel) to the antiparallel (parallel) configurations, between the upper and lower portions of the Co/Pd multilayers, were defined as *H*_*AP*_ (*H*_*P*_), respectively. Plots of the spin-flip fields for the 3.0-nm-thick W-capped SAF as a function of the annealing temperature are shown in Fig. 3(b). As evident in this figure, the spin-flip fields decreased rapidly at 350 °C from the as-deposited states. Nevertheless, the AFC trend with sharp spin-flip was maintained. As mentioned in Fig. 2(d), these results seem to be closely related to the enhancement in the PMAs generated by the crystal structure ordering during annealing. However, higher annealing temperatures led to only a slight decrease in the spin-flip fields. This implies that after reaching a certain crystal structure ordering at a proper annealing temperature, the IEC and PMA features of the W-capped SAF remain unaffected and sustain the enhanced annealing stability. Figure 3(c) plots the summarized *H*_*ex*_ and the calculated -*J*_*iec*_ of W-capped SAFs as a function of the W capping layer thickness at various annealing temperatures. Detailed individual M-H loops and summarized *H*_*ex*_, *K*_*eff*_ · *t*_*FM*_, and -*J*_*iec*_ values are given in Figure S4 and Table S2 of Supporting Information, respectively. As seen in this figure, a rapid decrease in the *H*_*ex*_ and -*J*_*iec*_ values were observed for all of the samples annealed at 350 °C. However, only a slight decrement appeared when the annealing temperature was increased further, regardless of the *t*_*W,Cap*_. For example, the *H*_*ex*_ value of the 3.0-nm-thick W-capped SAF decreased from 2.97 kOe to 2.11 kOe at 350 °C; it was nearly saturated, corresponding to a saturated -*J*_*iec*_ value of approximately 0.35 erg/cm^2^. Even though much effort has been dedicated towards understanding the origin of the observed anomalous behavior of *H*_*ex*_ and -*J*_*iec*_ value, clear role of W capping layer thickness was not verified at this moment. Thus, we propose that the W-capped SAF structure with ultrathin capping layer thickness (*t*_*W,cap*_ = 1.0 and 2.0 nm) may suffer from unnecessary damage during deposition or annealing process, leading to unstable IEC features. However, the proper W capping thickness in the range of 2.5 ≤ *t*_*W,cap*_ ≤ 3.5 nm seems to provide the formation of appropriate and dense thin film, corresponding to the most stable IEC features upon annealing. On the other hand, a relatively thick W capping layer (*t*_*W,cap*_ = 4.0 and 5.0 nm) may induce rough interface arising from bombardment effect during deposition, resulting in the relatively weak IEC features and the presence of different spin-flip shapes, as shown in the M-H loops of as-deposited samples. In addition, a relatively large amount of W atom may accelerate the inter-diffusion event upon annealing, thus affecting the IEC collapse at an annealing temperature of 425 °C. Therefore, the optimized W thickness was experimentally determined to be 3.0 nm, which exhibited the relatively high *H*_*ex*_ and -*J*_*iec*_ values and most stable IEC features upon annealing due to the formation of appropriate and dense film.

### Structural analysis

**Figure 4:**
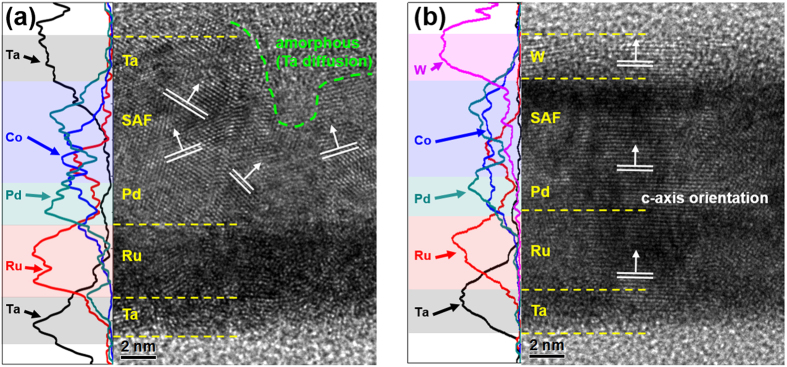
Cross-sectional HR-TEM images and corresponding EDS line profiles obtained for (**a**) Ta- and (**b**) W-capped SAFs for comparison. Both samples were annealed at 400 °C for 1 h under 3 Tesla.

A closer microstructural investigation of Ta- and W-capped SAFs was carried out using HR-TEM and the corresponding EDS line profiles for each element distribution map. The EDS line profiles were recorded with a scanning transmission electron microscope (STEM) mode over the same area that was used for HR-TEM imaging. Figure 4(a,b) present cross-sectional HR-TEM images of 3.0-nm-thick Ta- and W-capped SAFs annealed at 400 °C, respectively. As seen in this figure, the Ta- and W-capped SAFs displayed quite different crystal patterns after higher temperature annealing, despite the fact that identical Ta/Ru/Pd seed layers were employed for both SAFs. As shown in Fig. 4(a), the Ta-capped SAF showed a disoriented poly-crystalline structure without preferential orientation. The Ta capping layer and upper Co/Pd multilayer portion of the Ta-capped SAF had amorphous phases. An unclear interface between the Co/Pd multilayers and the Ta capping layer was also observed. This structural feature may indicate the presence of intermixing caused by thermally-activated Ta diffusion, which occurs during high temperature annealing. The EDS line profile for Ta (black line) verified possible Ta diffusion towards the upper portion of Co/Pd multilayers. The occurrence of the disoriented poly-crystalline structure can also be seen in the Co line profile. Detailed observations of the Co distribution in the SAF region also suggested the formation of non-uniform Co/Pd multilayers because Co/Pd multilayers, consisting of equi-atomic Co (0.3) and Pd (0.3), exhibited different atomic distributions in the Co (blue line) and Pd (cyan line) profiles. In contrast, the W-capped SAF exhibited single-crystal-like *c*-axis crystal features with uniformly well-aligned configurations, as evident in Fig. 4(b). Our previous observations, gleaned from W/CoFeB/MgO frames, provided evidence of superior annealing stability up to 425 °C. This was accomplished by suppressing inter-diffusion during annealing^27^. The difference in the structural and magnetic features between the Ta- and W-capped SAF is likely to arise from inherent characteristic of W capping layer. At first, the cohesive energy difference between Ta (8.1 eV/atom) and W (8.9 eV/atom) must be considered. Slightly higher cohesive energy of W capping layer may allow for suppressing inter-diffusion event of W atoms toward the SAF region upon annealing. As seen in the W (magenta line) profile, the atomic distribution of W layer was mainly detected from the initial capping layer region. In addition, the Co and Pd line profiles of the W-capped SAF displayed analogous atomic distributions, reflecting the proper formation of the Co/Pd multilayer structure, even after higher annealing temperatures was carried out. It is widely believed that the presence of strong PMA in a Co-based multilayer is linked with the formation of highly ordered crystal structure upon annealing^31^,^32^. In addition, the AFC behavior based on IEC is closely associated with the presence of smooth interface between the Co/Pd and Ru spacer layer^33^. Therefore, the single-crystal-like *c*-axis crystal features observed in the W-capped SAF, along with uniformly well-aligned structural configurations may lead to the strong PMA and AFC features. Closer analysis of the Co line profile revealed the presence of Co atoms in the Pd seed layer in both the Ta- and W-capped SAF frames; this may be the result of intermixing upon the annealing process. However, inter-diffusion does not appear to affect the magnetic crystalline structures of W-capped SAFs. It is widely believed that the crystal structure ordering of Co/Pd takes places at higher annealing temperatures. Thus, high temperature annealing may allow for the diffusion of Co towards the Pd seed layer region, reflecting the presence of additional CoPd layer inside the Pd seed layer region. In addition, as illustrated in the Fig. 2 and S2 of Supporting Information, the occurrence of near-zero remanence phenomenon at zero field might be linked with the observations of SAF frames consisting of two ferromagnetic layers with equal magnetic moments^8^,^33^,^34^. Therefore, we expect that additional CoPd seems to be created upon annealing.

### CoFeB reference layer

**Figure 5:**
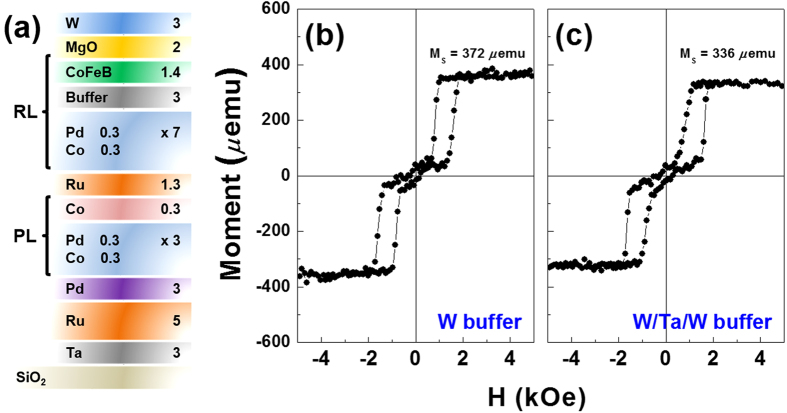
(**a**) Schematic illustration of the conventional CoFeB reference layer junction with SAF frames using W and W/Ta/W buffer layers. Representative M-H hysteresis loops of (**b**) W-buffered and (**c**) W/Ta/W-buffered RLs after annealing at a temperature of 425 °C.

To extend the applicability of the Co/Pd SAF frame as a pinning layer, two stacked frames were also constructed as follows: subs./Ta (3)/Ru (5)/Pd (3)/[Co (0.3)/Pd (0.3)]_3_/Co (0.3)/Ru (1.3)/[Co (0.3)/Pd (0.3)]_7_/W (3)/Co_20_Fe_60_B_20_ (1.4)/MgO (2)/W (3) and subs./Ta (3)/Ru (5)/Pd (3)/[Co (0.3)/Pd (0.3)]_3_/Co (0.3)/Ru (1.3)/[Co (0.3)/Pd (0.3)]_7_/W (1.2)/Ta (0.9)/W (0.9)/Co_20_Fe_60_B_20_ (1.4)/MgO (2)/W (3). Both frames were annealed at 425 °C. Figure 5(a) shows the representative schematic for both frames, where the former frame is referred to as the W-buffered RL and the latter is referred to as the W/Ta/W-buffered RL. In an attempt to use a material with a higher boron affinity feature of Ta than W^35^, an ultrathin Ta layer was intentionally inserted in the W/Ta/W-buffered RL frame. As shown in Fig. 5(b), the W-buffered RL displayed a typical M-H loop with near-zero remanence at the zero field as well as sharp spin-flip behavior. The *M*_*S*_ value of the W-buffered RL was 372 *μ*emu, while the W-capped SAF was 321 *μ*emu. The difference between the two frames was 51 *μ*emu. The increment of the *M*_*S*_ was caused by the adjacent CoFeB layer in the W-buffered RL. However, since no additional spin flip in the M-H loop of the W-buffered RL containing a CoFeB layer was detected, the adjacent CoFeB layer and upper part of the Co/Pd multilayers (acting as a single RL) were antiferromagnetically coupled, as shown in Fig. 5(a). Furthermore, sharp switching from the parallel state to the antiparallel state between the RL and pinning layer layers was observed up to 425 °C, verifying that the W layer may be a suitable BL for real device applications. However, the W/Ta/W-buffered RL yielded a slightly canted M-H shape and a small increment in the *M*_*S*_ (of about 15 *μ*emu), where the M-H loop shape seems to reflect the spin-flop mechanism^28^,^29^. Such features are likely caused by thermally-activated Ta atoms in the Ta layer drifting into the CoFeB layer or into Co/Pd multilayers at higher annealing temperatures; this leads to degradation of the PMA and IEC features.

## Discussion

In this work, we address thermally-strong PMA and IEC features of W-capped SAFs and W-buffered RLs by incorporating a W layer. These materials satisfy the well-known criteria of the key demands for device application. The use of a proper W layer provided wide-ranging plateau behavior with a sharp spin-flip mechanism up to 425 °C. This thermally stable IEC features upon annealing seems to be directly linked to the ability to suppress intermixing event due to the formation of appropriate and dense W layer, along with a high cohesive energy. Our structural analysis findings provided evidence of the formation of a single-crystal-like *c*-axis oriented crystalline structure after annealing at high temperatures. Annealing process-induced diffusion of Co atoms into Pd seed layer led to the formation of additional CoPd, resulting in the near-zero remanence AFC behavior at zero-field. This result seems be quite agreeable with the observations of SAF frames consisting of two ferromagnetic layers with equal magnetic moments or thicknesses. In addition, the SAF/W/CoFeB reference layer exhibited quite stable PMA and IEC features upon annealing without unnecessary another spin-flip. Thus, we anticipate that the ability to improve the thermal stability via incorporation of a proper W layer will spur progress in the development of practical applications, even though the origin of these improved features is not yet clarified at this moment.

## Methods

Co/Pd multilayer-based SAF frames were prepared on thermally-oxidized Si (001) substrates by utilizing DC/RF-magnetron sputtering systems at room temperature. The base pressure was less than 5 × 10^−8^ Torr. The working pressure was fixed to 5 × 10^−3^ Torr with an Ar flow rate of 20 sccm. Co/Pd multilayers were deposited at a relatively high working pressure of 6.8 × 10^−3^ Torr with an Ar flow rate of 30 sccm to create dense films. The deposition rates for each layer were carefully controlled to be less than 0.1 Å/sec. After deposition, post annealing was employed in the temperature range of 350 to 425 °C for 1 h in vacuum conditions below 10^−6^ Torr within the presence of a 3 T perpendicular magnetic field. Magnetic properties of SAFs and related RLs were analyzed by utilizing a vibrating sample magnetometer (VSM, LakeShore 7410). M-H hysteresis loops were recorded at room temperature with external magnetic field sweeping in the range of 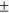
5 kOe applied in the direction of normal to the sample plane. Structural observations were taken via high-resolution transmission electron microscopy (HR-TEM, JEOL JEM-2100 F) and energy dispersive X-ray spectroscopy (EDS).

## Additional Information

**How to cite this article**: Lee, J.-B. *et al.* Thermally robust perpendicular Co/Pd-based synthetic antiferromagnetic coupling enabled by a W capping or buffer layer. *Sci. Rep.* **6**, 21324; doi: 10.1038/srep21324 (2016).

## Supplementary Material

Supplementary Information

## Figures and Tables

**Table 1:** Summarized *H*_*ex*_, *K*_*eff*_ · *t*_*FM*_ and -*J*_*iec*_ values of Pd, Ta, Ru, and W-capped SAFs at various annealing temperatures.

Sample	as-depo.			350 °C annealed			400 °C annealed		
	*H*_*ex*_	*K*_*eff*_ ·*t*_*FM*_	-*J*_*iec*_	*H*_*ex*_	*K*_*eff*_ ·*t*_*FM*_	-*J*_*iec*_	*H*_*ex*_	*K*_*eff*_ ·*t*_*FM*_	-*J*_*iec*_
	(kOe)	(erg/cm^2^)		(kOe)	(erg/ cm^2^)		(kOe)	(erg/ cm^2^)	
Pd-capped	2.39	0.63	0.66	1.29	0.63	0.25	-	0.63	-
Ta-capped	2.64	0.61	0.65	0.76	0.62	0.20	-	0.62	-
Ru-capped	2.57	0.61	0.65	0.86	0.61	0.22	-	0.61	-
W-capped	2.97	0.63	0.66	2.11	0.65	0.43	1.83	0.65	0.38
