# Supplementary material for: Thermally robust perpendicular Co/Pd-based synthetic antiferromagnetic coupling enabled by a W capping or buffer layer
Source: Sci Rep. 2016 Feb 18;6:21324. doi: 10.1038/srep21324 (PMC4758064; doi:10.1038/srep21324)
Supplement: Supplementary Information [file srep21324-s1.doc]

Supporting Information

Thermally robust perpendicular Co/Pd-based synthetic antiferromagnetic coupling enabled by a W capping or buffer layer

Ja-Bin Lee^1^, Gwang-Guk An^1^, Seung-Mo Yang^1^, Hae-Soo Park^1^, Woo-Seong Chung^2^,

and Jin-Pyo Hong^1,*^

^1^Novel Functional Materials and Device Lab., Research Institute of Convergence of Basic Science, Department of Physics, Hanyang University, Seoul, 133-791, South Korea

^2^Nano Quantum Electronics Lab., Department of Electronics and Computer Engineering, Hanyang University, Seoul, 133-791, South Korea

^*^jphong@hanyang.ac.kr


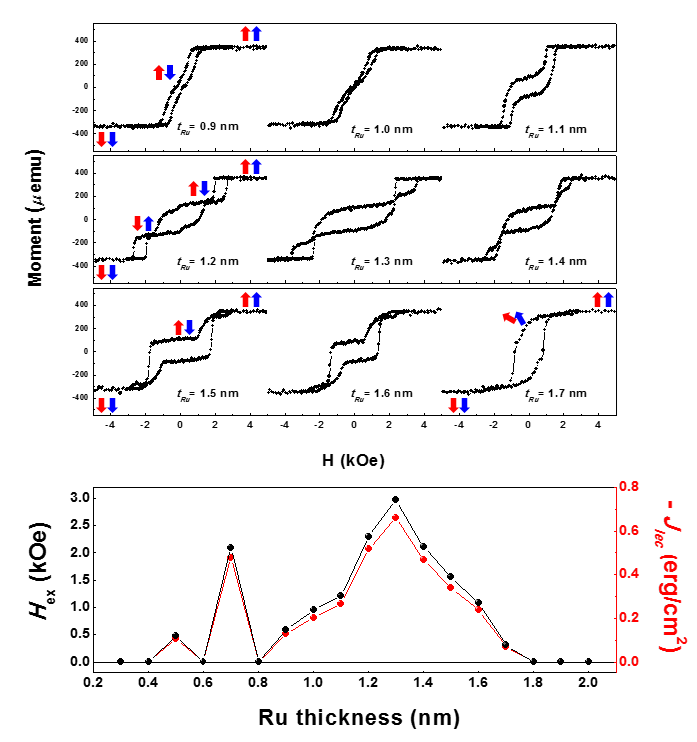


**Figure S1.** Individual M-H hysteresis loops and the *H_ex_* and calculated -*J_iec_* values of the Pd-capped SAF as a function of the Ru spacer layer thickness (ranging from 0.9 nm to 1.7 nm). Red and blue arrows in the figures represent the magnetization direction of the upper (repetition number = 7) and lower (repetition number = 3) portions of Co/Pd multilayers, respectively.


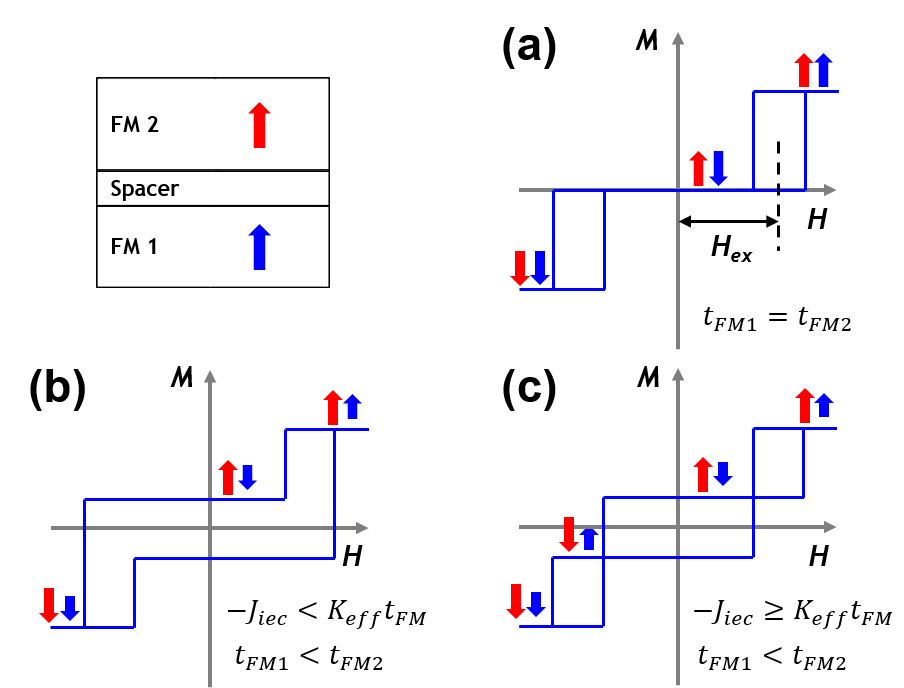


**Figure S2.** Schematic illustrations of various spin-flip mechanisms. (a) Typical M-H loop shape of SAF structure consisting of two ferromagnetic layers with equal magnetic moments or thicknesses, exhibiting zero remanence in AFC region. (b) Wide-ranging plateau appears in M-H loop of SAF structure consisting of two ferromagnetic layers with different magnetic moments or thicknesses. (c) Another spin-flip of two ferromagnetic layers with head-to-head and tail-to-tail magnetic configuration appears when the interlayer exchange coupling strength (-*J_iec_*) overcomes the areal effective perpendicular magnetic anisotropy energy (*K_eff_* · *t_FM_*).


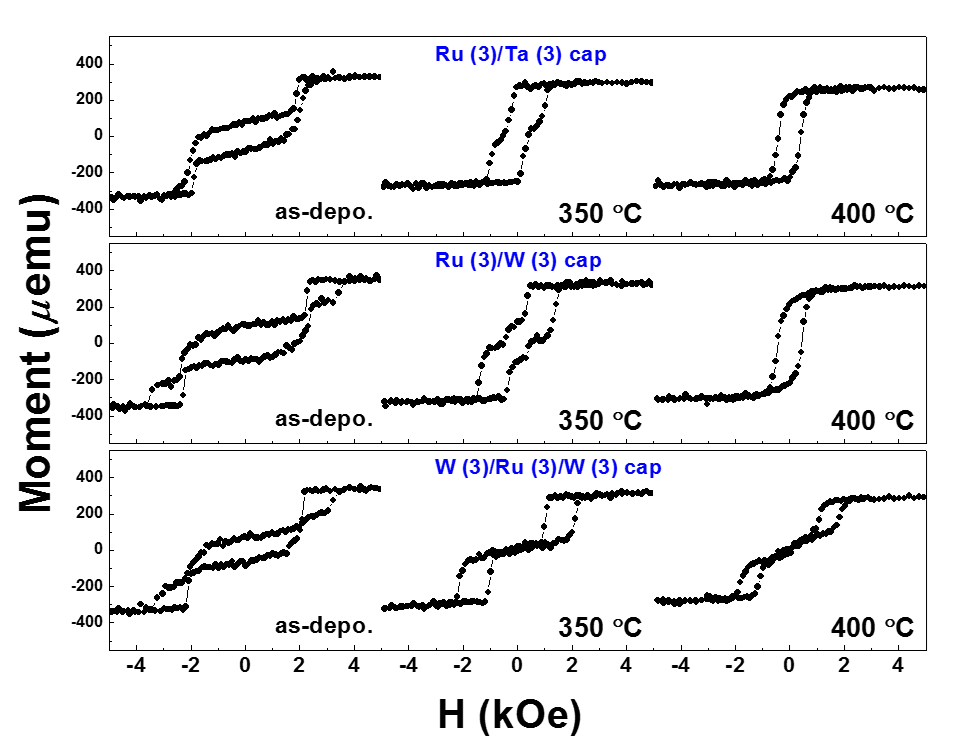


**Figure S3.** M-H hysteresis loops of SAFs with various stacked Ru/Ta (top), Ru/W (middle), and W/Ru/W (bottom) capping layers. The samples were annealed at 350 ^o^C and 400 ^o^C for 1 h under 3 Tesla.


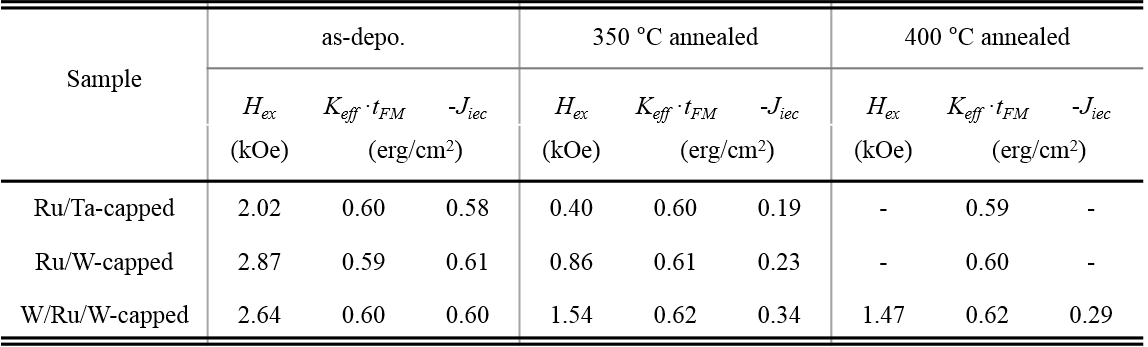


**Table S1.** Summarized *H_ex_*, *K_eff_* · *t_FM_* and -*J_iec_* values of Ru/Ta, Ru/W, and W/Ru/W-capped SAFs at various annealing temperatures.


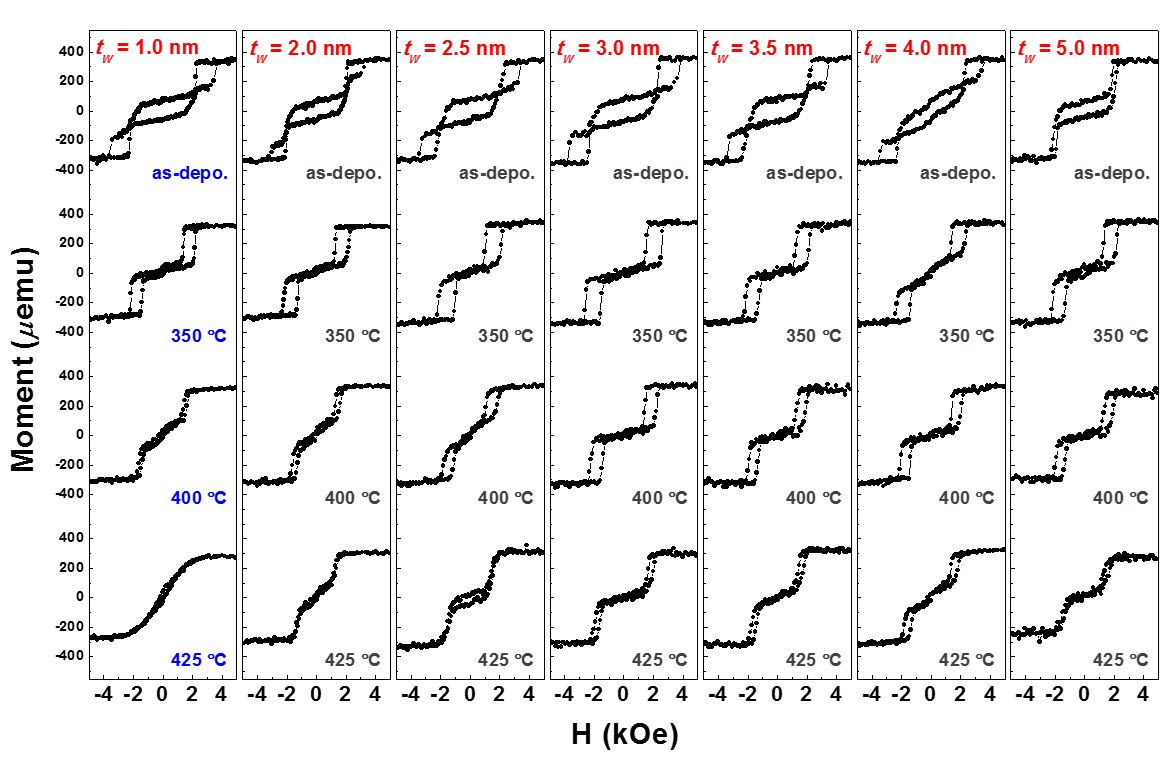


**Figure S4.** Individual M-H hysteresis loops of W-capped SAFs as a function of the W capping layer thickness (ranging from 1.0 to 5.0 nm). The samples were annealed at various temperatures ranging from 350 to 425 ^o^C for 1 h under 3 Tesla.


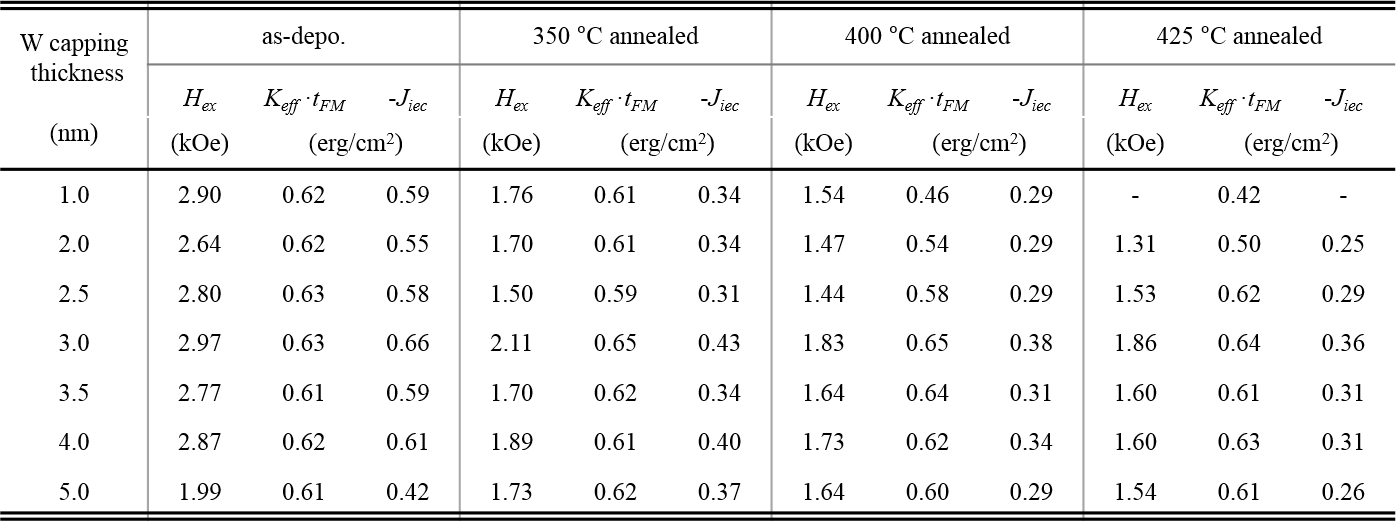


**Table S2.** Summarized *H_ex_*, *K_eff_* · *t_FM_* and -*J_iec_* values of W-capped SAFs as a function of the W capping layer thickness (ranging from 1.0 to 5.0 nm). The samples were annealed at various temperatures ranging from 350 to 425 ^o^C for 1 h under 3 Tesla.


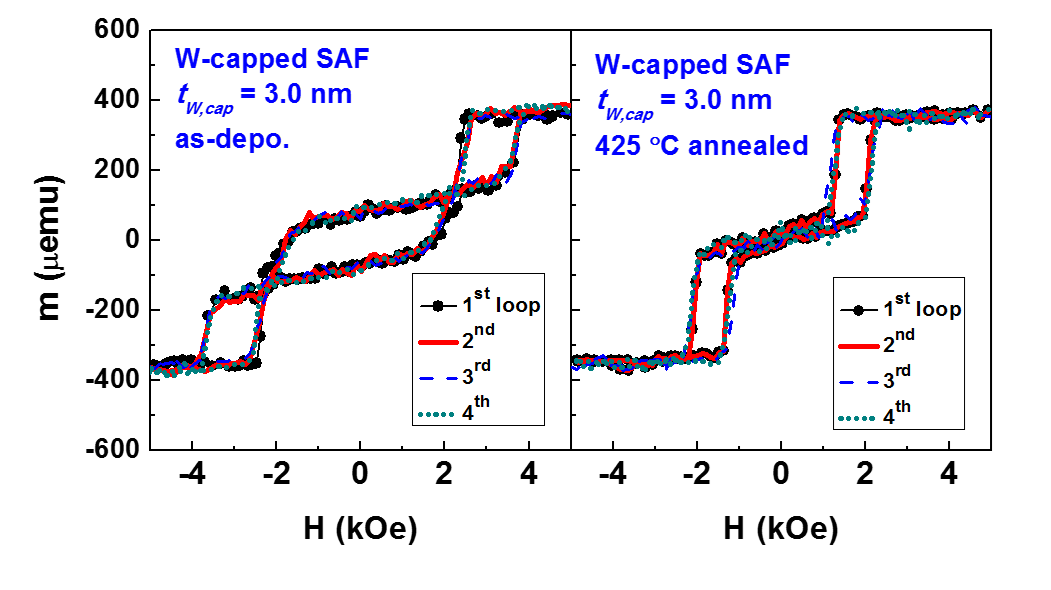


**Figure S5.** M-H hysteresis loops with isothermal four-cycles of W-capped SAF with *t_W,cap_* = 3.0 nm. The samples were annealed at 425 ^o^C for 1 h under 3 Tesla.


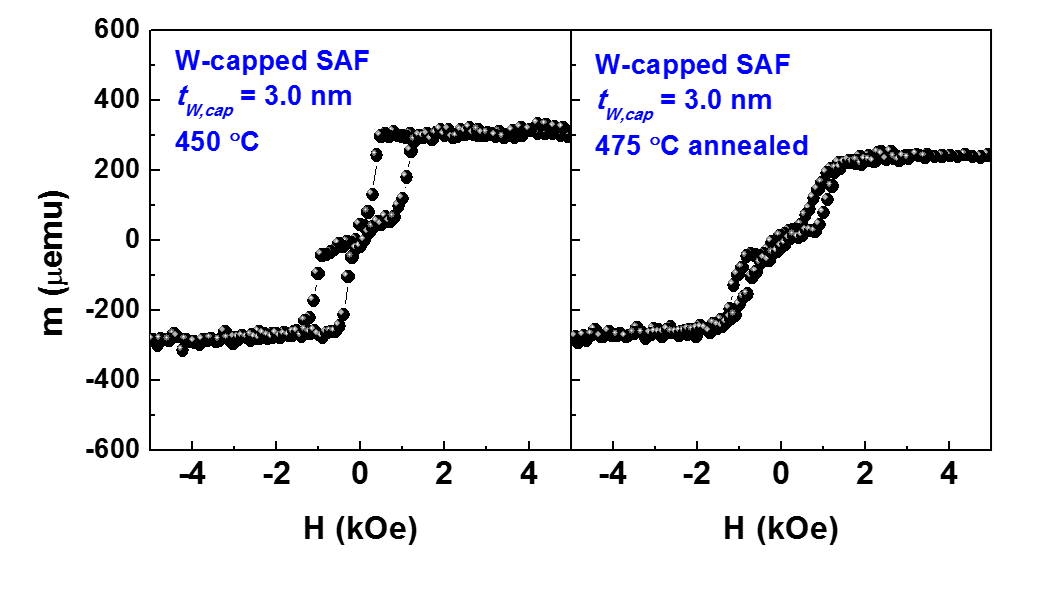


**Figure S6.** M-H hysteresis loops of W-capped SAF with *t_W,cap_* = 3.0 nm. The samples were annealed at 450 and 475 ^o^C for 1 h under 3 Tesla.
